# Neural mechanisms underlying touch-induced visual perceptual suppression: An fMRI study

**DOI:** 10.1038/srep37301

**Published:** 2016-11-22

**Authors:** Masakazu Ide, Souta Hidaka, Hanako Ikeda, Makoto Wada

**Affiliations:** 1 Developmental Disorders Section, Department of Rehabilitation for Brain Functions, Research Institute of National Rehabilitation Center for Persons with Disabilities, 4-1, Namiki, Tokorozawa-shi, Saitama, 359-8555 Japan; 2 Japan Society for the Promotion of Science (JSPS), Kojimachi Business Center Building, 5-3-1, Kojimachi, Chiyoda-ku, Tokyo, 102-0083, Japan; 3 Department of Psychology, Rikkyo University, 1-2-26, Kitano, Niiza-shi, Saitama, 352-8558 Japan

## Abstract

Crossmodal studies have demonstrated inhibitory as well as facilitatory neural effects in higher sensory association and primary sensory cortices. A recent human behavioral study reported touch-induced visual perceptual suppression (TIVS). Here, we introduced an experimental setting in which TIVS could occur and investigated brain activities underlying visuo-tactile interactions using a functional magnetic resonance imaging technique. While the suppressive effect of touch on vision was only found for half of the participants who could maintain their baseline performance above chance level (i.e. TIVS was not well replicated here), we focused on individual differences in the effect of touch on vision. This effect could be suppressive or enhancement, and the neuronal basis of these differences was analyzed. We found larger inhibitory responses in the anterior part of the right visual cortex (V1, V2) with higher TIVS magnitude when visuo-tactile stimuli were presented as spatially congruent. Activations in the right anterior superior temporal region, including the secondary somatosensory cortical area, were more strongly related to those in the visual cortex (V1, V2) with higher TIVS magnitude. These results indicate that inhibitory neural modulations from somatosensory to visual cortices and the resulting inhibitory neural responses in the visual cortex could be involved in TIVS.

Our perceptual systems continuously receive large amounts of sensory inputs from the surrounding environment. The brain automatically and efficiently integrates these inputs in order to establish coherent and robust percepts regarding the surrounding environment^1^. Studies on crossmodal interactions have consistently demonstrated enhancement/facilitatory effects for behavioral and neural responses^2^. For example, the perceived intensity of a visual stimulus is enhanced by a concurrent auditory stimulus^4^. The pooling of neural signals from these multimodal stimuli induces facilitatory neural responses in the superior colliculus (SC) of cats^5^,^6^. Crossmodal information is thought to induce activation not only in some higher sensory association cortices^7^ but also in primary sensory cortices^8^.

Some studies have also reported inhibitory neural interactions for crossmodal inputs. The pooling of neural signals from multimodal stimuli was also reported to induce inhibitory responses in the SC of cats^5^,^6^. Sounds or tactile stimulations were demonstrated to inhibit neural responses to light in the primary visual cortex and to suppress visually triggered behavioral responses in mice^9^. A human brain imaging study also showed that auditory or visual stimuli induced inhibitory responses in certain parts of the visual or auditory cortices, respectively^10^. Similarly, tactile stimulation to the hand was found to inhibit neural responses in visual cortical areas^11^,^12^.

Behavioral studies on crossmodal interactions revealed that faster behavioral responses were obtained for an attended modality than for an unattended one^13^,^14^. In relation to these, both facilitatory (indicated by positive blood oxygenation level dependent (BOLD) signal changes) (e.g., 15) and some inhibitory (indicated by negative BOLD signal changes) neural responses^10^,^11^,^12^ were associated with crossmodal attentional effects^16^. However, the inhibitory neural responses are also considered to be related to perceptual tasks and performances. In visuo-tactile interactions, tactile spatial discrimination performance improved when observers saw their body part (forearm) just before their judgments^17^. This phenomenon was assumed to be based on modulations in tactile neurons, including their receptive field sizes, by feedback projections from visual or visuo-tactile bimodal neurons. In fact, an event-related potential study demonstrated that brain activities in visual cortical areas could have modulatory effects on those in somatosensory cortical areas^18^. Another study reported that visual information of the participant’s body part induced suppressive effects when participants performed a tactile detection task or a tactile discrimination task for the stimuli presented above (10 times) the detection threshold^19^.

Recently, tactile suppressive effects on visual perception were also reported (touch-induced visual suppression; TIVS)^20^. This study showed that a tactile vibration degraded orientation discrimination performance for visual stimuli. TIVS occurred primarily when the visual stimuli were presented at the threshold contrast level for orientation discrimination and when the tactile and visual stimuli were spatially and temporally congruent. These findings suggest that the effect occurs not at an attentional level but at a perceptual processing level. A similar visual suppression effect was also reported for sounds^21^. While previous crossmodal studies have mainly indicated perceptual effects of visual information on other sensory modalities (i.e., visual dominance)^2^, TIVS demonstrates an opposite effect (from tactile to visual modality). Evidence regarding the neuronal bases of the suppressive behavioral effects of tactile modality on visual modality is necessary for further understanding the mechanisms of crossmodal interactions in the human brain.

In the present study, we introduced an experimental setting where tactile and visual stimuli were presented as spatially and temporally congruent so that TIVS^20^ could occur and investigated the neural changes related to the effect of tactile stimulation on visual perception using a functional magnetic resonance imaging (fMRI) technique. We measured behavioral performances prior to brain imaging and then estimated neural changes without any task. We presented a vibration to the observer’s index finger on the palm side of their left hand as a tactile stimulus and square grating patches as a visual target stimulus (Fig. 1A). We presented visual and tactile stimuli on the same (congruent in space) or opposite (incongruent in space) side of the space. The spatial congruency aspects enabled us to highlight the differences in neural responses between the conditions where visuo-tactile interactions could or could not occur^20^ wherein the visuo-tactile stimuli were presented in both conditions. We also introduced the condition in which no tactile stimuli were presented in order to highlight the effect of tactile presentation and visuo-tactile interactions. In addition to the comparisons of the experimental conditions, we also performed analyses estimating neural connectivity in order to investigate possible linkages of different sensory and/or association cortices underlying visuo-tactile interactions.

Neural changes related to crossmodal interactions have been reported in subcortical structures, sensory cortices and higher association cortices (e.g., 5–8). Based on aspects inherent to the TIVS phenomenon^20^,^21^ and the finding regarding neural changes related to tactile presentations^11^,^12^, we could predict that inhibitory responses occurred in some visual cortices, and that inhibitory modulations from somatosensory cortical areas and/or through lower subcortical structures or higher sensory association cortices would be involved in the inhibitory neural responses in visual cortical areas.

In the behavioral experimental session, participants were asked to perform a visual orientation discrimination task. The results indicate that TIVS was not well replicated here: The suppressive effect was observed in the half of participants who could maintain their baseline performance above chance level, whereas the opposite effect (enhancement) was observed in the other half of participants, whose baseline performance was below chance level. We therefore focused on the relationships between the magnitude of TIVS and changes in brain activities. We found larger inhibitory responses in the anterior part of the right visual cortical areas (V1, V2) with higher magnitude of TIVS when visuo-tactile stimuli were presented as spatially congruent. Moreover, activations in the right anterior superior temporal region, including the secondary somatosensory cortical area, were more strongly related to those in visual cortical areas (V1, V2) with higher magnitude of TIVS when visuo-tactile stimuli were spatially congruent. These results indicate that inhibitory neural modulations from somatosensory to visual cortical areas and the resulting inhibitory neural responses on visual cortical areas could be involved in the occurrence of TIVS.

## Results

### Behavioral experiment

Firstly, we ran the behavioral experiment in the fMRI scanner without running brain imaging scans. Then we asked the participants to observe the visual stimuli while ignoring the tactile stimuli without any task in the later brain imaging experiment. We employed this design to prevent any behavioral or neural artifacts related to motor and/or tactile responses that might be elicited by button presses from interfering with the targeted MR signal.

**Figure 1:**
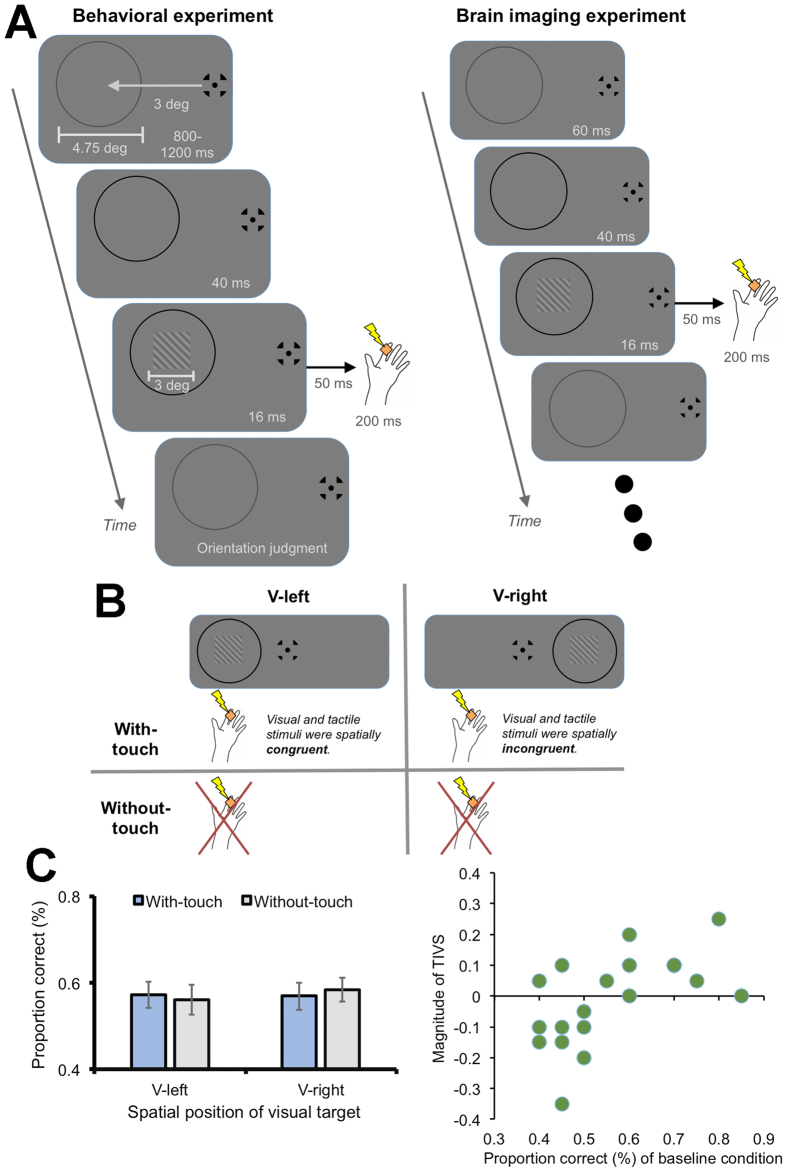
(**A**) Schematic illustrations of behavioral (left) and brain imaging (right) experiments. After the presentation of a fixation point and a gray ring, a visual target whose orientation tilted either left or right was presented at the center of the ring. The ring color was also changed from gray to black as the cue for the target onset. The tactile vibrations were presented to the participant’s left index finger 50 ms after the visual target presentation. Participants were asked to judge the target’s orientation in the behavioral experiment. (**B**) Schematic illustrations of experimental conditions. We set the four conditions depending on the presence of the tactile stimulus and spatial position of the visual target. (**C**) Results of the behavioral experiment. While significant effects were not observed for all participants (left panel), we focused on the relationship between the individuals’ magnitude of TIVS (right panel) and brain activities. In the left panel, error bars denote standard error of the mean. In the right panel, the magnitude of TIVS (positive values indicate stronger effects) was plotted against the proportion of correct responses for the V-left without-touch (baseline) condition. Hand images were produced by and used with permission from Shoko Yabuki.

The visual target stimuli consisted of square grating patches (3 × 3 deg, 0 deg of phase angle, 84% of Weber contrast) were presented at the left side of the fixation point (3 deg apart) against a gray background (0.83 cd/m^2^) for 16 ms. A gray ring (4.75 deg) was also presented around the target and its luminance was changed (from 0.21 to 0.03 cd/m^2^) to cue the onset of the target (Fig. 1A, left).

We initially introduced two adaptive staircase sequences (ascending and descending series) to estimate the spatial frequency value (cycles/deg) for 70.7% orientation discrimination performance for each participant (threshold estimation session). We asked the participants to judge whether the orientation of the target was perceived as tilting left (−45 deg) or right (+45 deg) without presenting tactile stimulus. In the next main session, we presented the targets for each participant with the estimated spatial frequency value at the left or right of the fixation point. After 50 ms, a vibration (200 Hz sinusoidal burst) was applied to the participants’ left index finger for 200 ms in order to effectively induce TIVS^20^. The participants were asked to make visual orientation judgments while ignoring the tactile stimulus. Trials without any tactile stimuli were also introduced. Thus, we had four conditions in total: The presentation or absence of presentation of the tactile stimulus at the visual target’s onset (with- or without-touch) and spatial position of the visual target (V-left or V-right) (Fig. 1B).

We calculated the proportion of correct responses in each condition for each participant (N = 19). A two-way repeated measures of analysis of variance (ANOVA) with the presentation of the tactile stimulus and spatial position of the visual target revealed no significant differences regarding main effects (*F*1,17 = 0.29, 0.01, *p* = .60, 92, respectively) or an interaction (*F*1,17 = 0.37, *p* = 0.55) (Fig. 1C, left). The reason why TIVS was not observed in all participants may be due to differences in performance levels in the baseline (V-left without-touch) condition (see the Discussion section for details). Suppressive behavioral performances were observed more strongly in participants who had a relatively high proportion of correct responses (>0.50) during the baseline condition, whereas the opposite tendency (enhancement) was observed in the remaining participants, whose baseline performance was relatively low (<0.50) (Fig. 1C, right). In later analyses of brain imaging data, we estimated the magnitude of TIVS (the values on the vertical axis in Fig. 1C, right) by subtracting the percent correct of the V-left with-touch condition from that of the V-left without-touch condition (positive values indicate the occurrence of TIVS), and we focused on the relationship between the individuals’ magnitude of TIVS and changes in brain activities.

### Brain imaging experiment

The brain imaging experimental session was performed after the behavioral experimental session. Each of the four conditions was tested twice in a random order (4 conditions × 2 times = 8 presentations) for 30 seconds. During each 30-second presentation, the visual stimuli with or without the tactile stimuli (350 ms of duration including the change in the ring color) repeatedly presented 15 times with 1.65 seconds of inter-stimulus interval (Fig. 1A, right). The orientation of the visual stimuli (left or right) was randomly assigned during the presentation.

First, we explored the neural changes for the with-touch condition in each visual stimulus position (*p* < .05, family-wise error (FWE) corrected at voxel level, more than 10 voxels). The Anatomy toolbox^22^ was used to examine whether activated brain areas were consistent with cytoarchitectonically defined areas (>5% of the cluster). Specifically, we focused on visual^23^,^24^,^25^ and somatosensory^26^,^27^ cortical areas, subcortical^28^,^29^,^30^,^31^,^32^ structures, and association cortices^33^,^34^,^35^ that are presumably related to crossmodal interactions^36^. In the V-left with-touch condition where the visual and tactile stimuli were spatially congruent, we found inhibitory responses for cortical areas assumed to correspond to the bilateral visual (V1, V2, V3v, V3d) cortical areas, right secondary somatosensory cortical area (S2), bilateral cerebellum, thalamus, and superior temporal gyrus (STG) against the blank period or the V-left without-touch condition (Supplementary Table 1; Supplementary Figure 1). In contrast, we found facilitatory responses for the V-right with-touch condition where the visual and tactile stimuli were spatially incongruent in the cortical areas corresponding to the left V1, right S2, STG, and intraparietal lobe (IPL) and sulcus (IPS) against the blank period or the V-right without-touch condition (Supplementary Table 2; Supplementary Figure 2). We did not observe differences in neural responses between the with-touch conditions for each visual stimulus position.

**Figure 2:**
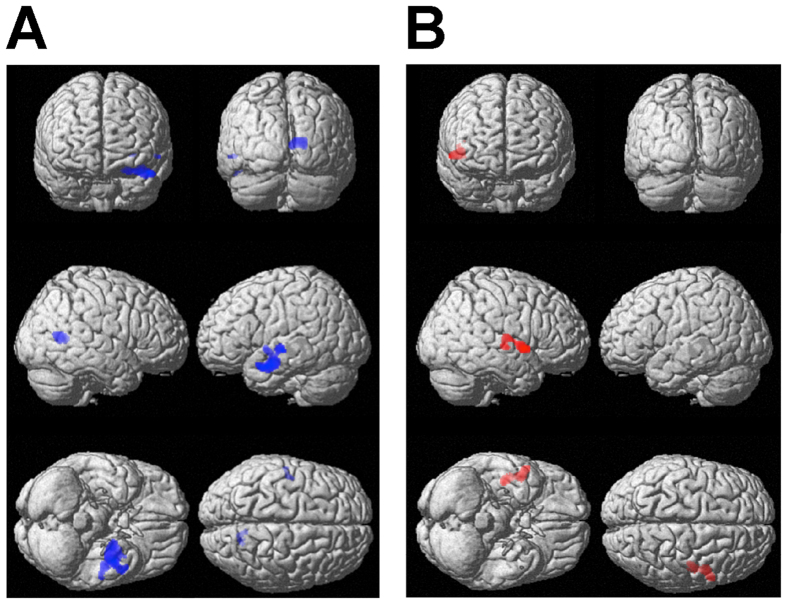
(**A**) Results of whole brain analyses between the V-left with-touch condition and the blank period along with the TIVS magnitude. (**B**) Results of the PPI analysis for the suppressed responses in the with-touch condition against the without touch condition in the V-left condition. The right visual cortical areas (V1, V2) were set as the seed regions. The analyses were performed uncorrected for multiple comparisons at voxel level (*p* < .001) and with correction for FWE at cluster level (*p* < .05).

We found inhibitory responses in visual and somatosensory cortical areas, lower subcortical structures, and higher sensory association cortices for the V-left with-touch condition against the blank period or the V-left without-touch condition. In order to further explore the inhibitory neural responses tightly related to TIVS, we performed the analyses with inclusion of the magnitude of TIVS as a covariate and investigated the effects of the covariate for the inhibitory neural responses in the V-left with-touch condition (*p* < 0.001, uncorrected for voxel level; *p* < 0.05, FWE corrected at cluster level). We found inhibitory responses in the areas assumed to correspond to the anterior part of the right visual cortical areas (V1, V2) against the blank period (Table 1; Fig. 2A, left). These results indicate that the higher the TIVS magnitude, the stronger inhibitory responses in the right visual cortical areas in the situation where visuo-tactile stimuli were spatially congruent so that TIVS could occur.

We further explored functional connectivity with TIVS magnitude by using a psychophysiological interaction (PPI) analysis. According to the results of the whole brain analysis that included the TIVS magnitude as the covariate, we set the cytoarchitectonically defined right V1 and V2^23^,^24^,^25^ cortical areas as the seed regions. Then, peak coordinates were identified in each participant for the V-left with-touch condition. The time course of the BOLD signal was extracted at the individually identified peak coordinates within a 6 mm sphere. We intended to focus on the connectivity firmly related to tactile processing and visuo-tactile interactions when the inhibitory neural responses occurred. Thus, we performed the PPI analysis with the psychological variables of without-touch – with-touch contrasts in the V-left condition along with the magnitude of TIVS. Significant interactions (*p* < 0.001, uncorrected for voxel level; *p* < 0.05, FWE corrected at cluster level) were observed for the right anterior superior temporal cortical area, which is assumed to correspond to S2 as well as the insula (Table 2; Fig. 2B). These results indicate that functional inhibitory connectivity between the somatosensory and visual cortical areas was stronger with higher TIVS magnitude.

## Discussion

In the present study, we introduced an experimental setting where TIVS could occur and investigated behavioral performances and neural changes related to the effect of tactile stimulation on visual perception in order to gain further understanding of the underlying mechanisms of visuo-tactile interactions. We manipulated both the presence of the tactile stimuli and the spatial congruency of the visual and tactile stimuli, and we measured the visual orientation discrimination performance and changes in brain responses with fMRI.

In the behavioral experiment, we observed perceptual suppressive effects of touch on vision only for half of the participants and the opposite effects (enhancement) for the other half (i.e. TIVS^20^ was not well replicated here) (Fig. 1C, right). We initially estimated the spatial frequency value at a 70.7% discrimination level for each participant in the threshold estimation session, after which the visual target was presented at the estimated threshold level for orientation discrimination in the main session. However, differences in performance levels during the baseline (V-left without-touch) condition were observed in the main session. Almost half the participants (N = 9) kept their performance above chance level (50%) and the average baseline performance was around the estimated threshold level. TIVS was observed for these participants (Supplementary Figure S3). However, the proportion of correct trials for the baseline condition was below chance level (50%) for the other half of the participants (N = 10). An enhancement effect rather than TIVS appeared for them (Supplementary Figure S3). A previous study investigated the effects of visual observation of the participant’s body part on tactile perception. The study demonstrated that the visual observation suppressed tactile discrimination performance when the tactile stimuli were presented at largely above the detection threshold^19^. On the contrary, the visual observation was reported to enhance discrimination performance for the tactile stimuli presented at the detection threshold level. In the present study, the participants whose baseline performance was below chance level were considered to have difficulty in detecting the visual target due to the weak magnitude of the stimuli. Similar to the previous findings^19^, therefore, the individual differences in detection level or perceived intensity for the visual target stimuli may have affected the visuo-tactile interaction (suppression or enhancement) in the current study. While investigations regarding the differences in the baseline performance are beyond the scope of this study and should be tackled in the future, we did, in the current study, investigate the neural changes related to visuo-tactile interactions underlying TIVS by focusing on the individual differences in TIVS magnitude as a covariate.

In the brain imaging experiment, the participants were exposed to the visual stimuli with or without the tactile stimuli. In the V-left with-touch condition where the visuo-tactile stimuli were spatially congruent so that TIVS could occur, inhibitory responses were observed for all participants against the blank period and V-left without-touch condition (Supplementary Table 1; Supplementary Figure 1). The cortical areas showing inhibitory responses corresponded to the bilateral visual cortical areas (V1, V2, V3v, and V3d), left S2, and bilateral cerebellum. In addition to S2, the cerebellum was also reported to be involved in the percept and discrimination of tactile inputs^37^,^38^. Inhibitory responses were also detected in subcortical structure and association cortices involved in crossmodal interactions (thalamus, STG, SPL)^36^. Inhibitory neural responses (i.e., negative BOLD signal changes) are assumed to be related to suppressive sensory processing^10^,^11^,^12^,^39^. On the other hand, when the stimuli were presented in a spatially incongruent manner (V-right with-touch condition), the cortical area related to tactile processing (S2) as well as the visual cortical area (V1) was found to be activated against the V-right without-touch condition (Supplementary Table 2; Supplementary Figure 2). These results could simply verify that our tactile stimuli were enough to induce activation in the corresponding cortical areas, and some visuo-tactile facilitatory effects could occur. It has been suggested that both bottom-up^40^, including subcortical sensory processes^41^, and top-down processes from association cortices^7^,^8^ are involved in crossmodal processing. In line with these ideas, the results observed in the V-left with-touch condition suggest that the inhibitory neural responses in the visual cortical areas and inhibitory modulations from somatosensory cortical areas, lower subcortical structures, and higher sensory association cortices may be involved in visuo-tactile interactions in the situation where TIVS could occur.

With regard to the inhibitory responses in the V-left with-touch condition against the blank period and V-left without-touch condition, we investigated the areas firmly related to TIVS by focusing on TIVS magnitude as the covariate. We found inhibitory responses in the anterior part of the right visual cortical areas (V1, V2) against the blank period along with the magnitude of TIVS (Table 1; Fig. 2A). Further, we explored the areas assumed to be functionally connected to the right visual cortical areas (V1, V2). Significant interaction was observed in the inhibitory responses of the V-left with-touch condition against the V-left without-touch condition along with the magnitude of TIVS. The detected region was the right S2 as well as the insula (Table 2, Fig. 2B), which was also considered to be involved in tactile processing^42^,^43^. These results indicate that neural activations in the cortical areas related to somatosensory and visual processing were primarily involved in TIVS. In the current study, we introduced the paired presentation of relatively weak visual stimuli (70.7% threshold level for orientation discrimination and 16 ms of duration) and a clearly perceptible tactile stimulus (200 ms of duration). It has been reported that unimodal presentation of tactile stimuli induced inhibitory neural responses in visual cortical areas^11^,^12^. In line with these findings, we could consider that tactile stimulation induced the inhibitory modulations from the somatosensory to visual cortical areas and consequently triggered the inhibitory responses in the primary visual cortical area in the current experimental setting.

Some studies have investigated neural changes related to visuo-tactile attentional enhancement effects. For example, a study^15^ reported that a tactile stimulus, which was spatially congruent with a visual one, induced facilitatory neural responses in the visual cortical area and the IPL, while spatially incongruent stimuli induced inhibitory responses in visual cortical areas. The main difference between these previous findings and the current findings is the visibility/reliability of the visual stimulus. The current study presented the visual stimuli at discrimination threshold level to induce crossmodal perceptual suppression. The previous study, on the other hand, presented a clearly visible stimulus in order to introduce a situation where attentional crossmodal enhancement effects could occur. Another study^16^ reported that attention to visual or auditory stimuli induced inhibitory neural responses in the unattended modalities (auditory or visual cortical areas, respectively). While this previous study presented the attentional cue 1000–1500 ms before the target onset in order to induce maximal attentional effects, the current study presented the visuo-tactile stimuli in a temporally congruent manner. In addition, cortical areas related to attentional modulation, such as the IPL and IPS (e.g., 15), were not found for the inhibitory neural responses in the V-left with-touch condition. Thus, in line with behavioral evidence^20^,^21^, we could assume that attentional modulatory effects are not a key factor for TIVS.

The findings in the current study suggest that the crossmodal inhibitory neural responses, specifically inhibitory neural modulations from somatosensory and the resulting inhibitory neural changes in the visual cortical areas, are involved in the occurrence of TIVS. The functional role of cortical inhibitory responses could be to maintain or enhance the processing of stimulated modality inputs by inhibition of other inputs at cortical^44^ and subcortical^45^ levels. Thus, one possible functional role of TIVS would be the establishment of coherent and robust percepts of our surrounding environment by suppressing weak or unreliable inputs as perceptual noise^20^,^21^. However, we could not draw firm conclusions because we observed TIVS only for half of the participants (i.e. TIVS was not well replicated here), and the current findings are based on the analyses of the covariate effect. Future research should induce a reliable TIVS magnitude for all participants. For example, we could separately estimate and manipulate the target detection threshold (e.g., contrast/spatial frequency of the target) and target discrimination threshold (e.g., orientation of the target)^19^. It is also possible to adopt event-related or rapid block designs while controlling for artificial motor or tactile signals in order to investigate the neural changes firmly related to the occurrence of TIVS.

## Methods

### Ethics statement

The experimental procedures were approved by the local ethics committee of the Research Institute of National Rehabilitation Center for Persons with Disabilities and were performed in accordance with the approved guidelines and the Declaration of Helsinki. Informed consent was obtained from each participant before conducting the experiments.

### Participants and apparatus

Twenty-two people participated, but two were excluded due to a higher autistic trait (over cut-off score^46^) or higher schizotypal trait (exceeding the upper boundary of 95% confidence interval) as measured by the Japanese version of the Autism Spectrum Quotient^46^,^47^ or the Japanese version of the Schizotypal Scale^48^,^49^, respectively. In addition, a participant was also excluded from the analysis because a cyst in the right posterior parietal cortex was detected. Thus, 19 participants’ data were analyzed. They claimed to have normal or corrected-to-normal vision and normal hearing. The participants were naïve to the purpose of the experiment. The visual stimuli were presented from a projector (NEC MODEL GT1150) to a half mirror with a resolution of 1024 × 768 pixels and a refresh rate of 60 Hz with a linearized luminance profile. The viewing distance was 140 cm. Tactile stimuli were presented through a vibrator (Uchida Denshi, PTD-2010-1). A customized PC (DELL, DIMENSION 9100) and MATLAB (MathWorks, Inc.) with the Psychophysics Toolbox^50^,^51^ were used to control the experiment. An fMRI compatible keypad (Uchida Denshi, UDS-2012-4) was used to record responses. We confirmed that the onset of the visual and tactile stimuli was synchronized using a digital oscilloscope (OWON, PDS5022TFT). The experiments were conducted in a dark shield room, and the participants were instructed to lie down in the fMRI scanner with their head position fixed.

### Stimuli

For visual stimuli, a fixation point consisting of a bull’s-eye and crosshairs (0.3 × 0.3 deg; 0.03 cd/m^2^)^52^ and a gray ring (4.75 deg in diameter; 0.05 deg in width; 0.21 cd/m^2^) were presented on a gray background (0.83 cd/m^2^). The ring was presented at a position to the left of the fixation point at 3 deg of horizontal distance, and its color was changed from gray to black (0.03 cd/m^2^) during target presentations. We also presented square grating patches (3 × 3 deg, 0 deg of phase angle, 84% of Weber contrast) as the visual target for 16 ms at the center of the ring. The stripes of the target stimulus tilted either left (−45 deg) or right (+45 deg). For the tactile stimulus, a vibration (200 Hz) was presented for 200 ms.

### Behavioral experiment procedure

At the beginning of the experiment, we ran two adaptive ascending and descending staircase sequences with a “2-up, 1-down” rule. Since our visual presentation setup had a low and small range of luminance (from 0.03 to 1.70 cd/m^2^), we estimated a spatial frequency value (cycles/deg) for the 70.7% orientation discrimination performance for each participant (threshold estimation session). After presentation of the fixation point and the gray ring for 800–1200 ms (randomly assigned in each trial), the ring color changed from gray to black as the cue for target onset for 40 ms. Then, the visual target was presented. No tactile stimuli were presented during the session. We asked the participants to judge whether the orientation of the target was perceived as tilting left or right. The target’s orientation was randomly assigned in each trial. The target’s spatial frequency changed in response to the participant’s judgment (correct/incorrect) in each trial. While the descending series started from 2 cycles/deg frequency, the ascending series began from 12 cycles/deg. Each sequence was terminated when 15 response reversal points were obtained. The spatial values of the last 10 points of the two sequences or the descending sequence (depending on whether the sequences converged or not) were averaged to estimate each participant’s threshold, and the value was adopted as the target spatial frequency in the subsequent main session. The averaged spatial frequency values (SD) were 6.23 (1.91) cycles/deg.

In the main session (Fig. 1, left), the tactile stimulus was presented 50 ms after the visual target onset (with-touch condition) in order to effectively induce TIVS^20^. We asked the participants to judge the orientation of the target while ignoring the tactile stimulus. The condition without the tactile stimulus was also included (without-touch condition). In this condition, a blank period was presented for 50 ms after the visual target presentation. We presented the visual target left or right of the fixation. Therefore, we set four conditions in total: The presence of the tactile stimuli (with-/without-touch) and spatial position of the visual target (left or right) (Fig. 1B). We repeated each condition 20 times for 80 trials in total. The order of these conditions was randomly assigned in each trial and counterbalanced among the participants.

### Brain imaging experiment procedure

Each of the four conditions was tested twice in a random order (4 conditions × 2 times = 8 presentations) for 30 seconds. During each 30-second presentation, the visual stimuli with or without tactile stimuli were repeatedly presented 15 times. The duration of the visual and/or tactile stimuli presentation was 250 ms. Similar to the behavioral experiment session, the gray and black ring was presented for 60 and 40 ms, respectively, before the visual and/or tactile stimuli presentation. The inter-stimulus interval was 1.65 seconds. The orientation of the visual stimuli (left or right) was randomly assigned during the presentation. The participants were asked to observe the visual stimuli while ignoring the tactile stimuli without any task. Blank periods (30 seconds) with the presentation of the fixation point were interleaved between the presentations of each condition. The blank period was also applied at the initiation (90 seconds) and termination (60 seconds) of the session. The session was repeated two times so that each condition was presented four times in total. Except for these differences, the stimulus parameters were identical to those in the behavioral experiment.

### fMRI procedure and data analysis

We collected fMRI data by using a 1.5T-MRI system (Toshiba Medical Systems) with a 16-channel head coil (Toshiba Medical Systems, MJQH-127A). The parameters of the functional scans were as follows: TR = 2.5 seconds, TE = 40.0 ms, flip angle = 90°, spatial resolution = 3.9 × 3.9 × 3.9 mm, FOV = 25 mm, slice thickness = 6 mm, inter-slice gap = 2.0 mm, number of slices = 18. For each participant, 240 functional brain volumes were obtained (the first 12 volumes were discarded from the analysis). We also obtained a T1-weighted anatomical scan for each participant (TR = 2.75 ms; TE = 12 ms; flip angle = 90°; spatial resolution = 1.4 × 1.4 × 1.4; FOV = 35 mm; slice thickness = 4 mm; inter-slice gap = 1.0 mm; number of slices = 33).

We used SPM12 (Wellcome Department of Imaging Neuroscience, London, UK) with MATLAB for preprocessing and statistical analyses. After motion correction, the functional brain images of each participant were co-registered and normalized into the standard space defined by the Montreal Neurological Institute (MNI) template. Then, the images were smoothed with a 3D-Gaussian kernel (full width, half maximum = 8 mm).

First, activations in each condition and session were computed for each participant based on the general linear model. Each condition and session was separately modeled. All regressors were convolved with the canonical hemodynamic response function and included in the subsequent model, but the session factor was collapsed. Next, we performed a whole brain analysis based on all of the participants’ data with a repeated measures analysis of variance (ANOVA) design to consider the factors of condition and participant while collapsing the factor of the session.

We set the statistical contrast between the with-touch condition and blank period or without-touch conditions in each visual stimulus position. These analyses were performed using a 5% statistical significance level with correction for FWE at the voxel level. In addition, we performed analyses where each participant’s magnitude of TIVS was included as a covariate, and the effect of the covariate was estimated using an uncorrected 0.1% statistical level at voxel level and 5% statistical significance level with correction for FWE at cluster level. PPI analysis was also performed to investigate functional connectivity regarding suppressed responses in the congruent with-touch condition. We set the right V1 and V2^23^,^24^,^25^ cortical areas as the seed regions and identified peak coordinates in each participant based on the effects of interest contrast between the V-left with-touch condition and the blank period. Then, the time course of the BOLD signal was extracted at the individually identified peak coordinates within a 6 mm sphere. The significant statistical level for detecting the peak coordinates was set below uncorrected 0.5% for 12 participants with the exception of 2 participants at 0.8% and 5 participants at 2% due to a lack of significant coordinates. We performed the PPI analysis with the psychological variables of the V-left without-touch – with-touch contrasts along with the magnitude of TIVS in order to closely focus on the effects of the tactile stimulation and visuo-tactile interactions. Significant interaction areas were detected with a multiple regression design and with an uncorrected 0.1% statistical level at the voxel level and 5% statistical level with FWE correction at the cluster level.

The Anatomy toolbox 2.2 (ref. 22; http://www.fz-juelich.de/inm/inm-1/DE/Forschung/_docs/SPMAnatomyToolbox/SPMAnatomyToolbox_node.html) was utilized to determine whether activated brain areas were consistent with cytoarchitectonically-defined areas. We used the WFU PickAtlas toolbox (ref. 53; http://imaging.mrc-cbu.cam.ac.uk/imaging/MniTalairach) in order to convert the MNI spaces of the detected activation areas into Talairach spaces and to identify the Talairach labels of these areas.

## Additional Information

**How to cite this article**: Ide, M. *et al*. Neural mechanisms underlying touch-induced visual perceptual suppression: An fMRI study. *Sci. Rep*. **6**, 37301; doi: 10.1038/srep37301 (2016).

**Publisher’s note:** Springer Nature remains neutral with regard to jurisdictional claims in published maps and institutional affiliations.

## Supplementary Material

Supplementary Information

## Figures and Tables

**Table 1:** Statistical and locational information for inhibitory neural responses in the V-left with-touch condition against the blank period along with TIVS magnitude.

Region	Broadmann area		Talairach coordinates			Cluster size	T	Anatomically defined regions (% of the cluster)
			x	y	z			
Sub-gyral (Temporal Lobe)		L	−38	−9	−17	1474	5.40	
Parahippocampal gyrus			−26	−64	−13		4.59	
Insula	13		−45	−14	−2		4.52	
Posterior cingulate		R	20	−60	11	512	4.65	V1 (48.8)/V2 (13.9)
			9	−55	11		4.42	

The analyses were performed with uncorrected for multiple comparisons at voxel level (*p* < .001) and with correction for FWE at cluster level (*p* < .05).

**Table 2:** Statistical and locational information for inhibitory neural responses in the V-left with-touch condition against the V-left without-touch condition detected by the PPI analyses along with TIVS magnitude.

Region	Broadmann area		Talairach coordinates			Cluster size	T	Anatomically defined regions (% of the cluster)
			x	y	z			
Superior temporal gyrus	22	R	48	0	2	595	5.55	Secondary somatosensory area (9.8)
Insula	13		42	−14	9		4.93	
Insula	13		44	−15	−1		4.16	

The analyses were performed with uncorrected for multiple comparisons at voxel level (*p* < .001) and with correction for FWE at cluster level (*p* < .05).
