# Supplementary material for: Neural mechanisms underlying touch-induced visual perceptual suppression: An fMRI study
Source: Sci Rep. 2016 Nov 22;6:37301. doi: 10.1038/srep37301 (PMC5118811; doi:10.1038/srep37301)
Supplement: Supplementary Information [file srep37301-s1.docx]

## Supplementary Information

## Neural mechanisms underlying touch-induced visual perceptual suppression: An fMRI study

Masakazu Ide^12#^*, Souta Hidaka^3#^*, Hanako Ikeda^1^, and Makoto Wada^1^

1. Developmental Disorders Section, Department of Rehabilitation for Brain Functions, Research Institute of National Rehabilitation Center for Persons with Disabilities, 4-1, Namiki, Tokorozawa-shi, Saitama, 359-8555 Japan.
2. Japan Society for the Promotion of Science (JSPS), Kojimachi Business Center Building, 5-3-1, Kojimachi, Chiyoda, Tokyo, 102-0083, Japan.
3. Department of Psychology, Rikkyo University, 1-2-26, Kitano, Niiza-shi, Saitama, 352-8558 Japan.

# These authors equally contributed to the work.

*Corresponding authors:

Masakazu Ide

E-mail: ide-masakazu@rehab.go.jp

Developmental Disorders Section, Department of Rehabilitation for Brain Functions, Research Institute of National Rehabilitation Center for Persons with Disabilities, 4-1, Namiki, Tokorozawa-shi, Saitama, 359-8555 Japan

Souta Hidaka

E-mail: hidaka@rikkyo.ac.jp

Department of Psychology, Rikkyo University,

1-2-26, Kitano, Niiza-shi, Saitama, 350-8558 Japan.

Supplementary table 1. Statistical and locational information for neural responses related to the V-left with-touch condition (the visual and tactile stimuli were spatially congruent).

| Region | Broadmann area |  | Talairach coordinates | | | Cluster size | T | Anatomically defined regions  (% of the cluster) |
| --- | --- | --- | --- | --- | --- | --- | --- | --- |
|  |  |  | x | y | z |  |  |  |
| *Blank > V-left with-touch* | | | | | | | | |
| Culmen |  | L | -4 | -65 | -9 | 1807 | 7.90 | Left cerebellum (52.4) /  Right cerebellum (27.5) /  Right V2 5.9 |
|  |  | R | 10 | -55 | -9 |  | 6.99 |  |
|  |  | L | -12 | -50 | -11 |  | 6.48 |  |
| Parahippocampa gyrus |  | L | -32 | -50 | -6 | 294 | 6.96 | V1 (8.5) |
| Middle occipital gyrus |  |  | -28 | -60 | 3 |  | 6.15 |  |
| Parahippocampa gyrus |  | L | -24 | -32 | -3 | 170 | 6.28 | Thalamus (parietal area) (24.1) / Thalamus (temporal area) (20.6) / Thalamus (visual area) (14.1) |
| Sub-lobar |  |  | -21 | -25 | -2 |  | 6.12 |  |
| Middle temporal gyrus |  | L | -51 | 1 | -19 | 238 | 6.19 |  |
| Superior temporal gyrus |  |  | -51 | 0 | -2 |  | 5.60 |  |
| Middle temporal gyrus |  |  | -50 | -3 | -12 |  | 5.52 |  |
| Insula |  | R | 36 | -3 | 14 | 147 | 6.22 | Secondary somatosensory area (37.4) |
|  |  |  | 44 | 0 | 8 |  | 5.63 |  |
| Paracentral lobule | 31 | R | 4 | -15 | 45 | 122 | 6.17 |  |
| Superior temporal gyrus |  | R | 53 | 13 | -13 | 111 | 5.69 |  |
| Extra-Nuclear |  | R | 15 | -48 | 15 | 93 | 6.18 |  |
| Sub-gyral (frontal lobe) | 6 | R | 20 | 0 | 53 | 76 | 5.83 |  |
| Middle frontal gyrus | 6 | R | 27 | -6 | 50 |  | 5.52 |  |
| Thalamus |  | L | -14 | -29 | 10 | 61 | 5.68 | Thalamus (temporal area) (100) |
| Inferior frontal gyrus |  | R | 39 | 27 | -13 | 56 | 5.75 |  |
| Cuneus | 18 | L | -3 | -72 | 17 | 52 | 5.60 | V2 (71.2) / V1 (21.2) / V3d (7.7) |
| Cuneus |  | L | -10 | -85 | 19 | 39 | 5.48 | V1 (38.5) |
| Middle temporal gyrus | 21 | R | 62 | -6 | -6 | 28 | 6.48 |  |
| Superior temporal gyrus | 22 | L | -48 | -12 | -1 | 27 | 5.48 |  |
|  |  |  |  |  |  |  |  |  |
| Thalamus |  | R | 15 | -21 | 3 | 20 | 5.67 | Thalamus (parietal area) (60.0) /  Thalamus (somatosensory area) (40.0) |
| Paracentral lobule |  | L | -8 | -18 | 42 | 19 | 5.46 |  |
| Insula |  | L | -39 | -14 | -3 | 13 | 5.38 |  |
| *V-left: Without-touch > With-touch* | | | | | | | | |
| Middle temporal gyrus |  | R | 62 | -4 | -7 | 298 | 6.72 |  |
| Superior temporal gyrus | 38 |  | 59 | 7 | -9 |  | 6.01 |  |
|  |  |  | 50 | 9 | -13 |  | 5.57 |  |
| Left cerebellum |  | L | 0 | -68 | -9 | 141 | 6.01 | Cerebellum (98.6) |
| Culmen |  |  | -3 | -59 | -5 |  | 5.57 |  |
| Middle temporal gyrus |  | L | -53 | 2 | -18 | 95 | 6.57 |  |
| Sub-gyral (temporal lobe) |  |  | -45 | -1 | -23 |  | 5.22 |  |
| Cuneus | 18 | R | 4 | -76 | 26 | 55 | 5.71 | Right V3d (61.8) / Left V3d (17.0) /  Superior parietal lobe (5.5) |
| Precentral gyrus |  | R | 42 | -12 | 42 | 41 | 5.56 |  |
| Precentral gyrus |  | R | 27 | -25 | 54 | 40 | 6.12 |  |
| Superior temporal gyrus | 38 | L | -50 | -2 | -10 | 27 | 5.54 |  |
| Lingual gyrus |  | R | 18 | -56 | -1 | 33 | 5.41 | V1 (27.3) / V3v (9.1) / V2 (6.1) |

The analyses were performed with correction for FWE at voxel level (*p* < 0.05). Clusters over 10 voxels were reported.

Supplementary table 2. Statistical and locational information for neural responses related to the V-right with-touch condition (the visual and tactile stimuli were spatially incongruent).

| Region | Broadmann area |  | Talairach coordinates | | | Cluster size | T | Anatomically defined regions  (% of the cluster) |
| --- | --- | --- | --- | --- | --- | --- | --- | --- |
|  |  |  | x | y | z |  |  |  |
| *V-right with-touch > Blank* | | | | | | | | |
| Sub-gyral (temporal lobe) |  | L | -33 | -63 | 3 | 119 | 6.48 |  |
| Middle occipital gyrus |  |  | -39 | -72 | 4 |  | 5.92 |  |
| Superior parietal lobule | 7 | L | -21 | -45 | 41 | 14 | 5.35 | Intraparietal sulcus (92.9) |
| *V-right: With-touch > Without-touch* | | | | | | | | |
| Insula |  | R | 45 | -19 | 18 | 484 | 7.52 | Secondary somatosensory area (72.5) / Intraparietal lobe (9.3) |
| Postcentral gyrus |  | R | 55 | -17 | 17 |  | 6.45 |  |
| Extra-nuclear |  | R | 26 | 18 | 5 | 115 | 6.55 |  |
| Sub-gyral (temporal lobe) |  | L | -32 | -62 | 7 | 68 | 6.47 | V1 (20.6) |
| Middle frontal gyrus |  | L | -27 | 35 | -4 | 46 | 5.89 |  |
| Inferior frontal gyrus | 44 | L | -59 | 15 | 16 | 31 | 5.99 |  |
| Superior temporal gyrus |  | R | 36 | -40 | 11 | 28 | 6.05 |  |
| Insula |  | R | 36 | -3 | 14 | 21 | 5.68 | Secondary somatosensory area (57.1) |
| Sub-gyral (frontal lobe) |  | R | 24 | -26 | 33 | 18 | 5.47 |  |
| Inferior frontal gyrus |  | L | -46 | 25 | 1 | 12 | 5.47 |  |

The analyses were performed with correction for FWE at voxel level (*p* < 0.05). Clusters over 10 voxels were reported.


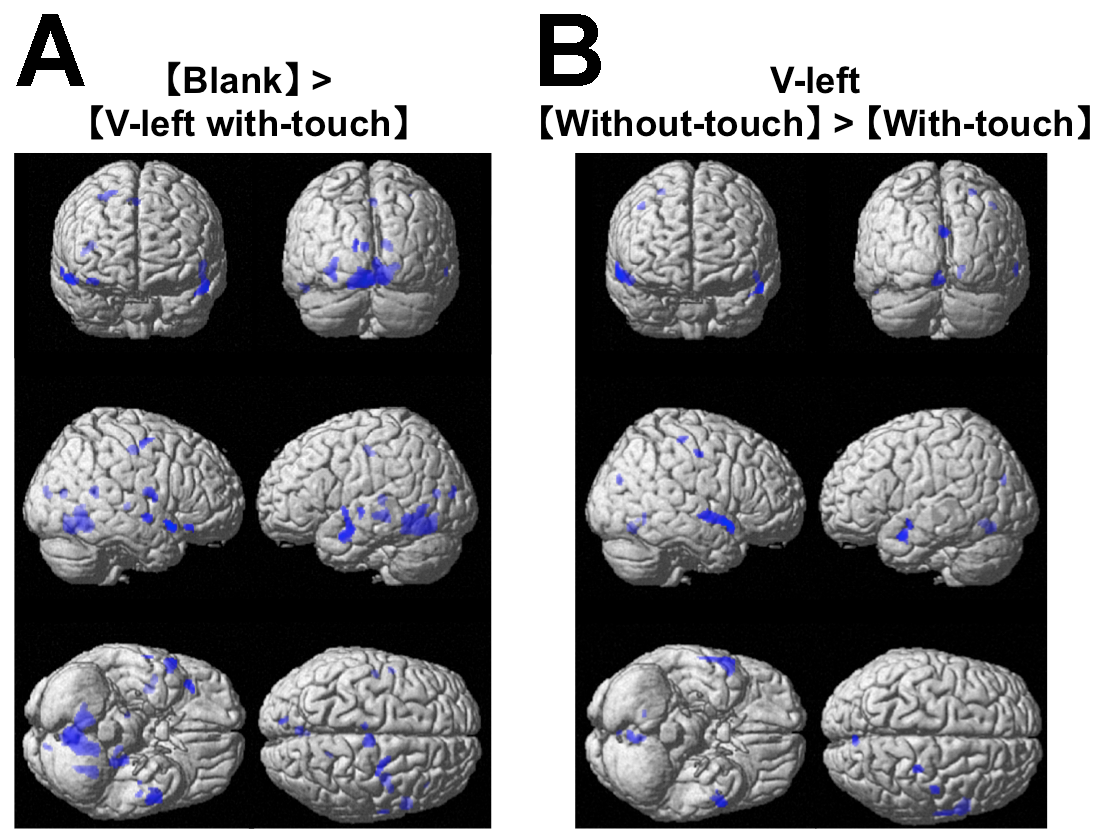


Supplementary figure 1. Results of whole brain analyses between the V-left with-touch condition (the visual and tactile stimuli were spatially congruent) and (A) the blank period or (B) the V-left without-touch condition (*p* < .05, FWE corrected for multiple comparison, more than 10 voxels).


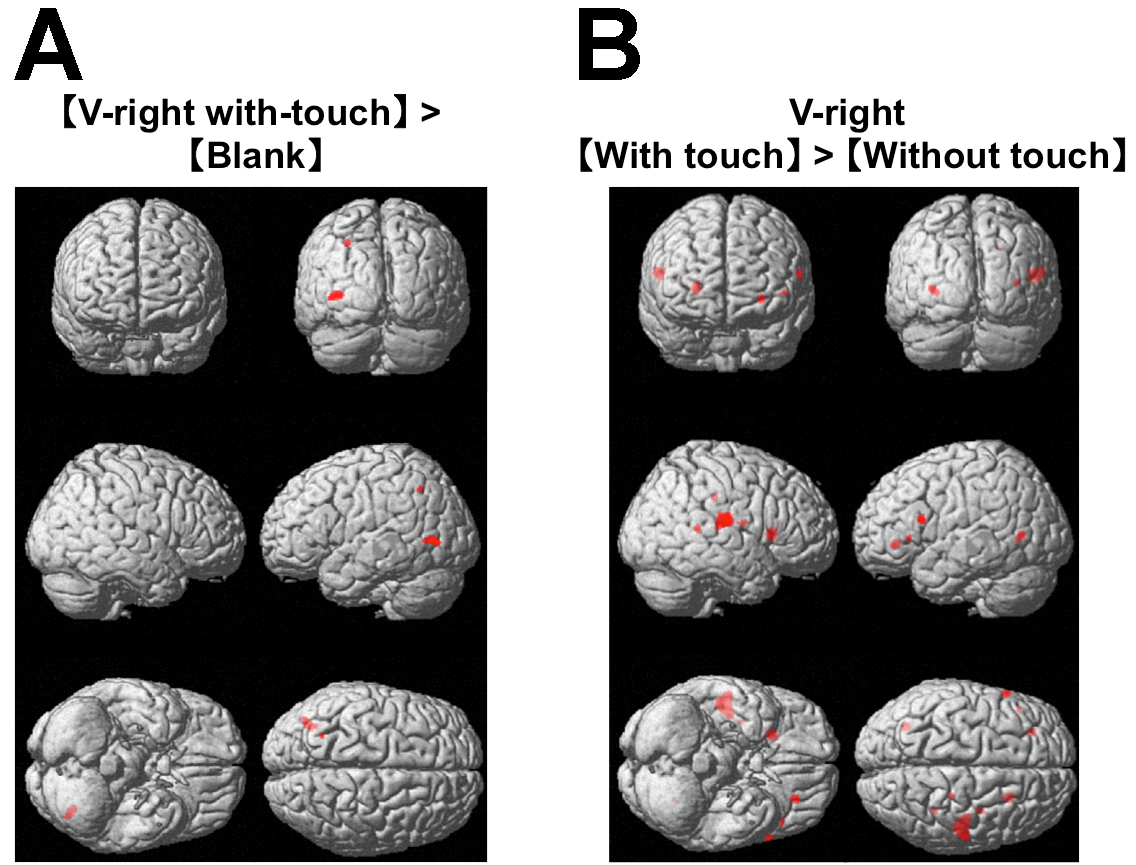


Supplementary figure 2. Results of whole brain analyses between the V-right with-touch condition (the visual and tactile stimuli were spatially incongruent) and (A) the blank period or (B) the V-right without-touch condition (*p* < .05, FWE corrected for multiple comparison, more than 10 voxels).


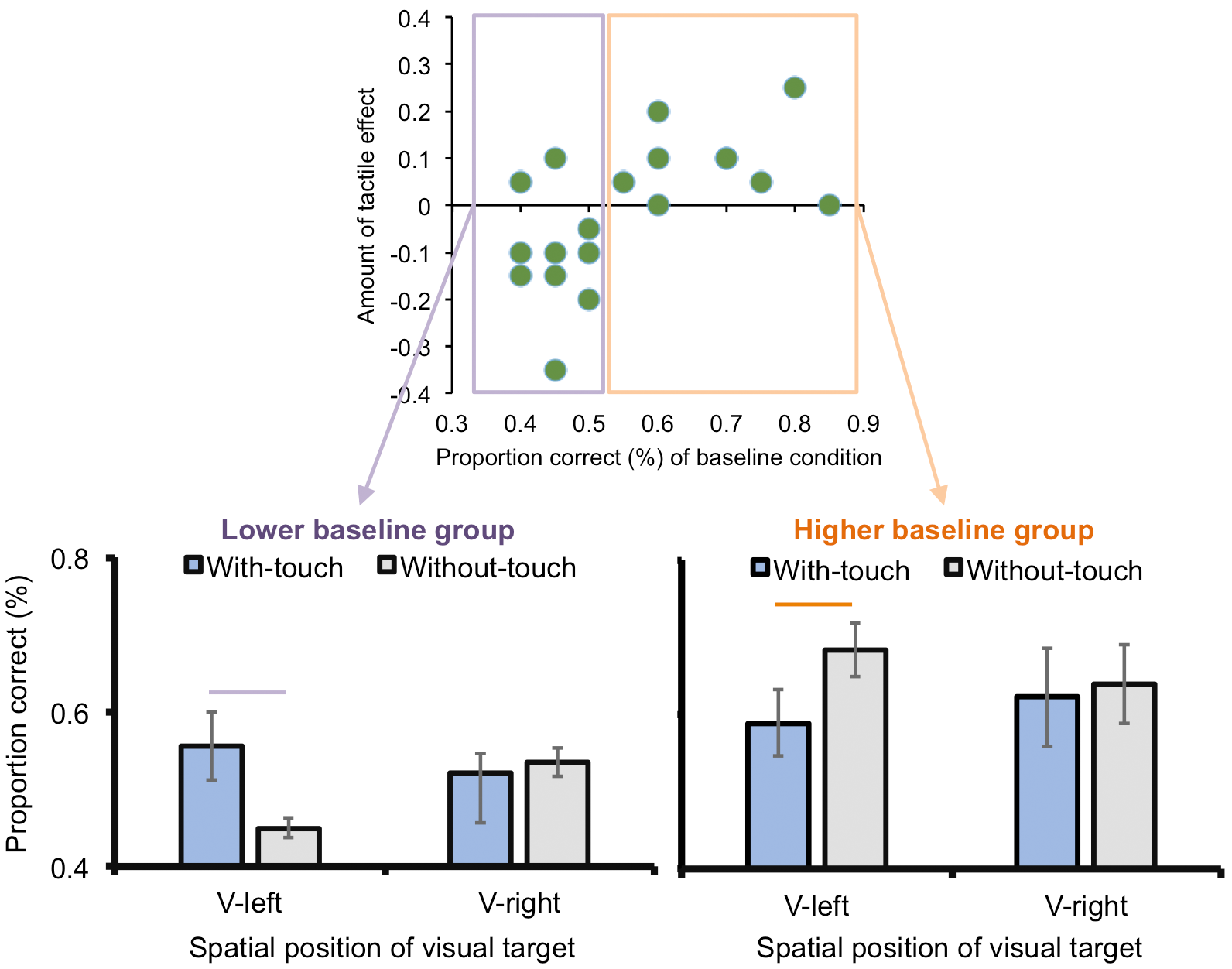


Supplementary figure 3. Results of the behavioral experiment divided by the participants’ baseline performances. The participants were separated into two groups based on the proportion of correct trials in the V-left without-touch condition. While we initially estimated the spatial frequency value at a 70.7% discrimination level for each participant, the proportion of correct trials for the V-left without-touch condition was below chance level (50%) for almost half of the participants (lower baseline group) and over chance level for the others (higher baseline group). A mixed design 3-way analysis of variance (ANOVA) with the group (between participants factor), the presence of the tactile stimulus, and the spatial position of the visual stimuli (within participants factors) revealed a significant interaction effect among all factors (*F*(1,17) = 6.98, *p* = .002). Simple simple main effects showed that the proportion of correct responses for the V-left with-touch condition (the visual and tactile stimuli were spatially congruent) was lower than that of the V-left without-touch condition in the higher baseline group (*p* = .034), whereas the lower baseline group showed an opposite tendency (*p* = .015). Error bars denote standard error of the mean.
